# Civility in Health Care: A Moral Imperative

**DOI:** 10.1007/s10730-022-09501-y

**Published:** 2022-12-22

**Authors:** Joel M. Geiderman, John C. Moskop, Catherine A. Marco, Raquel M. Schears, Arthur R. Derse

**Affiliations:** 1 Ruth and Harry Roman Emergency Department, Department of Emergency Medicine, Cedars-Sinai Medical Center, 8700 Beverly Blvd, Los Angeles, CA 90048 USA; 2 Department of Internal Medicine, Medical Center Blvd, Wake Forest University School of Medicine, Winston-Salem, NC 27157 USA; 3 Department of Emergency Medicine, Penn State Health – Milton S. Hershey Medical Center,, Penn State College of Medicine, 500 University Drive, Hershey, PA 17033 USA; 4 Department of Emergency Medicine, College of Medicine, University of Central Florida, 6850 Lake Nona Blvd, Orlando, FL 32827 USA; 5 Department of Emergency Medicine, Center for Bioethics and Medical Humanities, Medical College of Wisconsin, Kern Institute for the Transformation of Medical Education, M1100, 8701 Watertown Plank Road, Milwaukee, WI 53226-3548 USA; 6 General Internal Medicine, Wake Forest University School of Medicine, Medical Center Boulevard Winston-Salem, Winston-Salem, NC 27157 USA

**Keywords:** Civility, Disruptive Behavior, Professionalism, Violence, Codes of Conduct, Conflict Resolution, Ethics

## Abstract

Civility is an essential feature of health care, as it is in so many other areas of human interaction. The article examines the meaning of civility, reviews its origins, and provides reasons for its moral significance in health care. It describes common types of uncivil behavior by health care professionals, patients, and visitors in hospitals and other health care settings, and it suggests strategies to prevent and respond to uncivil behavior, including institutional codes of conduct and disciplinary procedures. The article concludes that uncivil behavior toward health care professionals, patients, and others subverts the moral goals of health care and is therefore unacceptable. Civility is a basic professional duty that health care professionals should embrace, model, and teach.

## Introduction

Desiderius Erasmus of Rotterdam, commonly known as Erasmus, is often credited with emphasizing the value of civility in human interactions. A brilliant and prolific Catholic scholar who lived during the Northern European Renaissance and the Reformation, he was a contemporary of Martin Luther. Erasmus often disagreed with Luther on matters of theology, but not disagreeably. He was willing to listen to Luther’s arguments and acknowledge them civilly.

As European civilization emerged from the Middle Ages, towns and cities grew and people lived in closer proximity to one another. What people did in public affected the lives of other human beings as never before. Erasmus wrote a book on etiquette for city children, *De civilitate morum puerilium* (1985). In it, he described what was expected of them from a European perspective as members of a civilized community. Erasmus’s treatise has been reprinted 130 times and translated from Latin into multiple languages. Norbert Elias, the influential sociologist and cultural historian renowned for his theories of civilizing and decivilizing processes, recognized the importance of Erasmus’s work in establishing social expectations and norms of civil behavior (2012).

The word ‘civility’ comes from the Latin *civilis*, meaning citizen, and is linguistically related to the word ‘city’. In Latin and later in French, the word referred to behavior befitting a citizen or a civilized person. Stephen L. Carter, author, scholar and Professor of Law at Yale University, relates a story of early train travel, where fellow travelers in very close spaces adopted rules to coexist with each other on long journeys. Among those rules were: “no singing, whistling, staring, loud talking or bodily expulsions. Alone among God’s creatures, human beings can make those choices, setting aside their own needs and desires for the sake of living in society with others” (1998, p. 4). Carter’s story illustrates the growing emphasis on behavior that respects the needs of one’s neighbors in civilized societies.

Immigrants to North America brought European ideas of civility with them. For example, in 1746, future American President George Washington, at age 16, wrote *110 Rules of Civility and Decent Behavior in Company and Conversation*, based on a set of rules composed by French Jesuits in 1595 (1926). In her analysis and defense of the moral virtue of civility, philosopher Cheshire Calhoun reports that “the nineteenth century saw a flood of etiquette manuals…[but] such manuals focused on deferential respect for rank and were not ultimately suitable to American egalitarian ideals” (2000, p. 258). American concepts of civility have thus evolved significantly with cultural and social changes.

According to an oft-repeated but perhaps apocryphal anecdote, an interviewer once asked Mahatma Gandhi, the great Indian leader and non-violence advocate, what he thought of Western civilization. According to the story, Gandhi cleverly replied, “I think it would be a good idea” (Quote Investigator, 2013). In this article, we examine the meaning and moral significance of civility and argue that civil behavior is not only a good idea, but also a moral imperative for health care professionals.

## What is Civility?

As noted above, civility in human behavior emerged as a norm in Early Modern Europe, and rules of civility have evolved with subsequent social changes. Our examination of civility in contemporary American health care, therefore, depends on a persuasive account of the meaning, scope, and moral significance of civility in America today. Calhoun offers such an account in her article “The Virtue of Civility” (2000), and this section will provide a brief summary of that account.

Calhoun argues that civility is a distinctive virtue that promotes morally significant goals of respect for other persons and maximization of utility. She acknowledges, however, that civility is not widely recognized as a basic moral virtue, and she offers three reasons for its relative neglect by moral philosophers. First, civility is closely linked with etiquette or good manners, and rules of etiquette, such as those that govern table manners, proper dress, and courteous driving, are viewed primarily as matters of social convention, not ethics. Second, socially established rules of civility may in fact reinforce unjust social arrangements, such as discrimination against women and minority groups. Third, the terms ‘civility’ and ‘incivility’ are used to refer to a vast scope of behaviors, and that wide scope may suggest that civility and incivility are not specific moral virtues and vices, but rather “catch-all” categories for all moral or immoral conduct.^1^ Regarding the first of the above claims, that civility is a matter of etiquette and not ethics, Calhoun responds that mannerly behavior is often distinctly moral, as, for example, considering others’ feelings, expressing gratitude, and respecting personal privacy. She also notes that civility is a morally significant mark of good citizenship, including obedience to the law and nonviolence. Regarding the second claim, that conventional rules of civility may reinforce social injustice, Calhoun acknowledges that, in our morally imperfect social worlds, conventions of civility can be unjust and may need to be resisted. In such worlds, she argues, we may face moral choices between *using* established social conventions to display our respect or tolerance for others, and *challenging* conventions that are not genuinely respectful or tolerant. Regarding the third claim, that civility is not a distinct moral virtue or type of moral behavior, but rather just a rough synonym for moral conduct, Calhoun asserts that civility has an essential feature that does distinguish it from other kinds of moral behavior. She describes that feature as follows:

In short, what makes being civil different from being respectful, considerate, or tolerant, is that civility always involves a display of respect, tolerance, or considerateness. Thus, civility is an essentially *communicative* form of moral conduct. In addition, because communicating our moral attitudes is central to civility, being genuinely civil—unlike, say, being genuinely considerate or genuinely tolerant—requires that we follow whatever the socially established norms are for showing people considerateness, tolerance, or respect. Only because there are such generally agreed upon, often codified, social rules for what counts as respectful, considerate, and tolerant behavior can we successfully communicate our moral attitudes toward others.. .. Similarly, incivilities draw on a common verbal and behavioral language for displaying disrespect, intolerance, or inconsiderateness (2000, p. 260).

Calhoun’s analysis thus explains the relationship between civility and etiquette, the moral significance of civil and uncivil behavior, and the distinctiveness of civility and incivility as types of conduct that *communicate* our moral attitudes toward other people.

Finally, Calhoun recognizes that civility is not an *absolute* value or duty; it is not, in other words, “the virtue of being nice no matter what” (2000, p. 267). To protect the practice of civil discourse in pluralistic societies, however, she limits the scope of permissible *uncivil* responses to actions whose intolerability is a matter of extensive social consensus, such as sexual harassment and racial discrimination. In the remainder of this article, we will rely on Calhoun’s general account of civility to examine its significance in the sphere of health care.

## The Role of Civility in Health Care

In the developed world, contemporary health care is a central social enterprise with a primary moral goal of the first order, namely, preserving and restoring human health and well-being. Achieving that beneficent goal depends on respectful, trusting, and cooperative relationships among health care team members, patients, and families. Positive relationships depend in turn on civil behavior. Multiple commentators offer evidence that civility in inter-professional and therapeutic relationships is crucial to patient safety, quality of care, teamwork, and job satisfaction (see, for example, McCue, 1982; Post & Weddington, 1997; Simpson & Grant, 1991; de Wall & Aureli, 1997; Marco, 1999; Oppel et al., 2019).

The human toll of uncivil behavior in health care settings is also morally significant, as incivility undermines patient outcomes, as well as professional morale, retention, and satisfaction. Some health care venues are particularly prone to lapses in civility. One example is the crowded hospital Emergency Department (ED), due to the stress of responding to the emergency treatment needs of multiple patients rapidly and effectively. Issues related to incivility in EDs have been reported globally, including in the US, Switzerland, the United Kingdom and Australia (Klingberg et al., 2018; Shetty et al., 2016; Bradley et al., 2015; Rosenstein et al., 2008). Cited examples of incivility include abrupt retorts or comments, unreasonable demands on colleagues, arbitrary assertions of power, shifting responsibility, blaming others, and hiding personal insufficiencies. Patients who require emergency care usually do not choose their ED treatment setting or their emergency physician. Because they are entering into a new therapeutic relationship with their patients, ED clinicians and non-clinical staff members can and should demonstrate attention and concern via respectful words and actions. Healthcare leaders and educators should promote efforts to demonstrate respect for patients and colleagues.

Despite the challenges of often stressful and high-stakes environments, we contend that civil conduct is essential to achieving the considerable benefits of health care and to demonstrating respect for other moral agents. We propose, therefore, that health care professionals owe a basic duty of civility in their interactions with patients, families, colleagues, and trainees. In the sections that follow, we will discuss health care professional interactions with each of these groups.

## Interactions with Patients and Families

In an article entitled “Etiquette-Based Medicine,” Kahn emphasizes the value of attentive and polite behavior by clinicians in communicating their respect for their patients. Kahn argues that these practices are an essential element of medical professionalism and of effective therapeutic relationships (2008). Physician Lucian Leape and colleagues have claimed that “being treated disrespectfully is devastating for patients” (Leape et al., 2012). Kahn’s and Leape’s claims provide insight into the moral significance of civil and uncivil human action.

Unfortunately, patients, family members, and other visitors sometimes exhibit uncivil behavior toward clinicians, including foul language, slurs, and threatening, aggressive, and violent behavior, including acts of criminal assault and battery (Gunderman & Gunderman, 2017). A recent study found that disruptive behavior is more common in EDs than five years ago (Marco et al., 2022). Acts of incivility, insults, threats, or violence are more likely to manifest in some healthcare venues than others due to the stress of acute illness or injury, mental illness, intoxication, substance use, unfamiliarity, disorientation, or delirium. Other transgressions include hateful language aimed at specific religious or cultural groups (Raman, 2017) and biased assumptions about a clinician’s training level or qualifications (Adaeze Okwerekwu, 2016).

Lack of an established therapeutic relationship in certain circumstances may also contribute to incivility experienced by health care professionals. P. M. Forni, a leading scholar and professor of Italian literature, co-founder of the Johns Hopkins Civility Project, and a prominent proponent of civility in modern American society, relates a humorous anecdote about two drivers who engage in “finger puppetry” after one cuts off the other in traffic, only to realize they know each other, after which they display embarrassment and revert to courtesy toward one another. Forni’s story illustrates the claim that incivility is easier when the person acting uncivilly thinks that he or she is “anonymous” to the recipient of her or his behavior (Forni, 2002).

Professional responses to uncivil behavior by patients and visitors should depend on the patient’s condition and the type of uncivil behavior. For patients whose lack of behavioral control is the result of acute psychosis, intense pain, extreme suffering, or severe delirium, clinicians may focus on providing treatments to calm the patient and enable him or her to regain control. These situations can be challenging, requiring clinicians to draw deeply on character traits of equanimity, tolerance, and courage to control their emotions in responding and attending to their professional duties (Hawking et al., 2017). Some patients who are aggressive or threatening require physical or chemical restraint for both their own safety and that of the treatment team (Kurter, 2019). Use of physical force on violent patients without their consent to protect them and professionals from harm is a clear example of recognition of the limits of civility in response to intolerable behavior.

When uncivil behavior by a patient prevents further benefit from a continuing therapeutic relationship, physicians can use a formal process to sever the relationship in some circumstances (Auckley, 2008). This option is not available to emergency physicians, trauma surgeons or others who are bound by professional and legal obligations, under the Emergency Medical Treatment and Labor Act (EMTALA), to determine whether an emergency condition exists, to treat or stabilize that condition, and if necessary to transfer the patient to a higher level of care (Emergency Medical Treatment and Labor Act, 1986). Determining the absence of an emergency medical condition to a degree of confidence sufficient to discharge the patient for uncivil behavior can be difficult to achieve (Bitterman, 2018). The physician, who is in a position of power in the doctor-patient relationship, must be careful not to let bias cloud judgment of a patient’s words or behavior. Nevertheless, we contend that patients who assault staff with slurs or threats or who engage in sociopathic, disruptive or violent behavior should be considered for removal or arrest. In these circumstances, physicians should be able to attest that an un-stabilized emergency medical condition as defined under EMTALA is not present. In other inpatient or outpatient treatment venues, physicians may need to make similar judgments about whether a disruptive patient’s medical condition permits removal of that patient from the venue without significant risk of harm. Physicians may consult risk management staff, legal counsel, or a hospital administrator, but consultation should not be required before taking this action.

Uncivil behavior by patients’ family members or other visitors is a somewhat different matter. Visitors may exhibit aggression or violence, and, if unchecked, may encourage others to exhibit this behavior as well. Distress and grief when a loved one dies or is severely ill is understandable, and health care facilities should respond appropriately to comfort patients and families in distress, but they should not tolerate aggressive behavior. In summary, health care facilities and individual clinicians should act to protect themselves, patients, colleagues, and visitors from abuse, assault or danger. Intervention by hospital security personnel or law enforcement officers may be necessary to assure safety and to facilitate removal of disruptive patients and visitors.

## Interactions with Colleagues

Over the past half century, interdisciplinary team-based care has become the dominant model of health care. Teams composed of physicians, nurses, advanced practice providers, social workers, physical therapists, pharmacists, and other health care professionals provide a wide variety of services for their patients. Securing the benefits of health care depends on effective collaboration among the members of health care teams, and that effectiveness depends in turn on civil interaction among the team members. As is the case with interactions with patients, however, health care professionals may act in uncivil ways toward professional colleagues. Uncivil behavior among professionals may include angry outbursts, derogatory comments, or subtle comments that denigrate other members of the team, as well as bullying or threats. Volz et al. characterize malicious behavior of emergency healthcare workers toward one another as “horizontal violence” and conclude that it is common (2017).

As leaders of health care teams, physicians bear distinctive responsibilities for relationships with patients and among colleagues. The Code of Ethics for Emergency Physicians, for example, recognizes both the value of inter-professional collaboration and the leadership role of physicians in these words: “though emergency physicians assume primary responsibility for patient welfare, emergency medicine is a team effort…physicians must coordinate the efforts of nurses and support staff” (American College of Emergency Physicians, 2017). As members of a specialty that opens its doors to all patients and interacts with all specialties, we believe that emergency physicians have a distinctive opportunity to set a high standard for civility in health care. To achieve that goal, emergency physicians must cultivate respectful relationships and eschew angry outbursts or assaultive behavior toward other health care professionals, trainees, patients, and visitors.

Physicians abandon their role as the moral leaders of health care teams if they exhibit disrespectful behavior toward other team members. In an article entitled “Barbers of Civility,” Klein and Forni cite the example of a surgeon who responds to being given a wrong instrument by a nurse by cursing at her and throwing the instrument (2011). Berating a nurse or resident and throwing instruments are destructive behaviors that were too long tolerated in the past and are not acceptable in any environment. Physicians should address errors or miscommunications civilly, and institutions and their leaders should not tolerate angry outbursts that humiliate or endanger the safety or dignity of others.

Professionals with differences in expertise, training, experience, and perspective may disagree on clinical issues (Graves, 1990). When such disagreements arise, colleagues may be tempted to attack the motives or abilities of others in disrespectful ways. To preserve respectful collegial relationships, health care professionals should seek to resolve disagreements by identification of shared goals and compromise solutions (Dubois, 2001). In some cases, they may negotiate solutions by focusing on sharing workload and decision-making in ways that do not jeopardize quality of care or patient safety. Colleagues should rely on civil interaction to settle disagreements and should do so privately, if possible.

## Interactions with Trainees

Based on polls of his students, P. M. Forni distilled the modern meaning of civility to four essential attributes (see Table 1) (2002). For many years, Forni taught and wrote about Italian fiction and poetry. He recounts his epiphany that if his students learned everything about Dante but nothing about civility, he would have considered himself a failure as a teacher. Like Forni, we believe that if clinicians know everything about a particular medical condition but do not act civilly and do not teach civility as a standard to students, they will be failures as practitioners and teachers of their professions.

**Table 1:** Forni’s attributes of civility

1. Civility is good
2. Civility is complex
3. Civility has to do with courtesy, politeness, and good manners
4. Civility belongs in the realm of ethics

Lecturing students about civility will be ineffective unless instructors also model civil behavior in their interactions with their students. Leape and colleagues, however, observe that “medical students too often suffer demeaning experiences at the hands of supervising faculty and residents,” and they assert that this behavior must change (2012). A 2018 report by the National Academies of Science, Engineering and Medicine (NASEM) singled out Surgery and Emergency Medicine as problematic areas during residency with regard to harassment of women due to their “hierarchical and authoritative workplaces” (Johnson et al., 2018; Marco et al., 2019). Hafferty and Franks famously pointed out the inconsistency between moral principles presented in the formal medical school curriculum and morally problematic behaviors and attitudes toward patients and trainees that are condoned by the “hidden curriculum” of medical socialization (1994).

Consider the following example: On a busy Monday night, while admitting a patient with sepsis who has received fluids, antibiotics and vasoactive drugs through a central line, an emergency physician may be annoyed by a resident or consultant who asks about a chart-accessible lab value. Although the physician may be inclined to reply with an uncivil comment, a polite and straightforward response is more likely to engender genuine learning, respect, cooperation, and collaboration.

Trainees, like all health care professionals, may make both medical and ethical choices that their instructors judge to be mistaken. In those situations, instructors may be tempted to respond by pointing out those errors in ways that publicly ridicule or humiliate the trainee, presumably with the intention of driving home the significance of the error in an especially powerful way. Are these situations justified exceptions to the responsibility to refrain from uncivil behavior? Robert Baker offers an example that appears to condone uncivil behavior in such a situation. In this example, a new intern who is severely pressed for time chooses to run a personal errand rather than transport a newly admitted premature infant for a screening CT scan. When the intern admits her decision to her attending neonatologist at patient rounds and suggests that it was reasonable,

the attending physician’s demeanor changed. She began to pace the staff room, hurling interrogatives at the intern. “Had you intended to inform us about this matter ... *Doctor?. . .* Do you intend to make a practice of abandoning your patients…*Doctor?. . .* And so it went, the attending physician pacing back and forth, pausing only to hurl a question at the intern. Each question ended with an emphatic pronouncement of the title, “Doctor.” The intern stood in stunned silence, seeming to shrink before our eyes, shuddering each time the attending pronounced the title, “Doctor.” The public excoriation of intern N before the assembled nurses and her fellow interns lasted almost two minutes—more than ninety very long seconds. (Baker, 2013)

Although he does not say so explicitly, Baker seems to approve of the attending’s behavior as a response to the intern’s failure to carry out her duty to her patient. We believe that it is essential for faculty to point out and discuss errors with their trainees, but we question whether public humiliation is an effective or appropriate way to pursue those discussions. Uncivil behavior by faculty role models is, in our view, more likely to inhibit genuine learning by instilling fear and creating barriers between teachers and learners. Those consequences may, in turn, interfere with teamwork and reduce the quality of patient care.

## Institutional Strategies to Promote Civility

Organizations suffer when people treat each other uncivilly across all levels (Porath & Pearson, 2013). To prevent the adverse consequences of uncivil behavior, many organizations strive to create and maintain a positive organizational culture that goes far beyond simple courtesy (Leape et al., 2012). Table 2 provides examples of uncivil behavior at the organizational level that can undermine a supportive culture.

**Table 2:** Uncivil organizational behaviors

· Neglecting to say please and thank you or respectful manner of address
· Texting, emailing and other forms of inattention during meetings
· Interrupting others
· Being late to meetings or leaving without explanation
· Keeping people waiting for appointments habitually and needlessly
· Taking too much credit for collaborative work
· Sending nasty or pithy emails
· Speaking condescendingly, including using a critical or abusive tone
· Condescending body language and mannerisms (eye rolling, smirking, etc.)
· Making disparaging remarks about colleagues’ care in the medical record.
· Placing derogatory or clinically irrelevant patient descriptors in the medical record, e.g., remarks about body habitus, personality, ethnicity, country of origin, or gender orientation

The Joint Commission (TJC) recommends that hospitals adopt policies for managing disruptive behavior (2021). Examples of disruptive behavior, a subset of uncivil behavior, include angry outbursts, rude acts, boundary violations, harassment, and profane or insulting language. Since the TJC recommendation, most hospitals have adopted codes of conduct (COCs). These are meant to regulate the conduct of physicians and other health care professionals, but they should ideally include everyone within a healthcare organization.

The effectiveness of such codes is dependent on enforcement. The star surgeon who brings in millions of dollars a year and demeans a nurse over a trivial matter, or the Board of Trustees member who curses at an ED triage nurse, should not be exempt from the expectation of professional and civil behavior. Hospital and medical staff leaders should model respectful behavior toward all members of the hospital community.

Remedies provided in institutional COCs should be proportional to the severity and frequency of the infraction. For minor, first time infractions, a conversation between the two parties involved should be encouraged, with a third party present, especially when more than one discipline is involved -- both to keep a record and to guard against power imbalances. Apologies may be useful in restoring collegial relationships.

If there is recidivism, or the complainant insists upon it, a formal COC investigation process should proceed. Physicians who are recidivists should be subject to stepwise discipline, which can result in loss of staff privileges. In many states, hospitals must report loss of privileges to the state medical board, which can limit future practice opportunities. Failure to respect COC requirements may also constitute harassment or discrimination and could lead to legal liability.

Some organizations find it better to have specific codes for specific populations; e.g., physicians, nurses, medical students, and administrators (Leape et al., 2012). These should mirror each other as much as possible. Unfortunately, a recent review concluded that organizational initiatives had limited influence on individual behavior (Gillen et al., 2017). A multifaceted approach is necessary, including education, open discussion, institutional initiatives, leadership support, policies and procedures, progressive counseling, consistent enforcement, and acceptance of individual responsibility (Maslach and Leiter, 2017; Howard and Embree, 2020; Clark and Kenski, 2017; Clark and Ritter, 2018; Di Fabio and Duradoni, 2019; Clark 2019).

Some hospitals post institutional statements of patient and health care worker rights and responsibilities. While difficult to enforce, especially before an emergency medical condition under EMTALA has been ruled out, such an approach, if crafted properly, clearly sets forth mutual expectations of behavior.

## Concluding Remarks

In this article, we have examined the concept and moral significance of civility in health care, and we have argued that health care professionals have a responsibility to act with civility. We conclude that civility in health care relationships, including observance of recognized norms of courtesy, kindness, toleration, and respect in speech and action, are fundamental moral responsibilities.

We view civility as a threshold below which professional behavior should never fall. Civility establishes a foundation for multiple other professional responsibilities, including beneficial care for patients, respect for patient choices, and stewardship of health care resources. Based on the arguments offered in this article, we conclude that civility deserves a place on the list of professional duties that health care professionals should teach and model.

## Footnotes

1 Calhoun offers the following expansive catalogue of uncivil conduct: “In addition to shoving, shouting, giving the finger, making insulting remarks, not waiting one’s turn in line, there are the incivilities of nosiness, bossiness, snobbishness, breaking appointments, overstaying visits, failing to offer thanks or apologies or responses to invitations, not reciprocating hospitality, hogging the road, littering, proselytizing, and offering unsolicited advice. Colonial American manuals on manners remind us of earthier incivilities such as returning half eaten food to communal dishes, scratching or revealing private parts, spraying spit while talking, and farting. And political philosophers include in their various lists of incivilities coercion, intimidation, harassment, violence, unrestrained pursuit of self-interest, the arbitrary exercise of power, disrespect for others’ rights and dignity, stating deep but unshared moral convictions, inattentively listening to another’s argument, intolerance, indifference to offense, voting on the basis of private preferences, expressing contempt for others’ life plans, engaging in vigilante justice, rioting, and not obeying the law” (2000, pp. 253–254).
